# ^18^F-FDG/^18^F-FES standardized uptake value ratio determined using PET predicts prognosis in uterine sarcoma

**DOI:** 10.18632/oncotarget.15127

**Published:** 2017-02-06

**Authors:** Makoto Yamamoto, Tetsuya Tsujikawa, Shizuka Yamada, Tetsuji Kurokawa, Akiko Shinagawa, Yoko Chino, Tetsuya Mori, Yasushi Kiyono, Hidehiko Okazawa, Yoshio Yoshida

**Affiliations:** ^1^ Department of Obstetrics and Gynecology, Faculty of Medical Sciences, University of Fukui, Fukui, Japan; ^2^ Biomedical Imaging Research Center, University of Fukui, Fukui, Japan

**Keywords:** ^18^F-FES PET, ^18^F-FDG PET, uterine sarcoma, prognostic prediction

## Abstract

We investigated whether 16α-[^18^F]-fluoro-17β-estradiol (^18^F-FES) and ^18^F-fluoro-deoxyglucose (FDG) uptake measured using positron emission tomography (PET) predicted prognosis in 18 patients with different histological subtypes of uterine sarcoma. Standardized uptake values (SUVs) and ^18^F-FDG/^18^F-FES SUV ratios were determined, and their correlations with progression-free (PFS) and overall survival (OS) were examined. Ten patients died from local recurrence or metastasis, and one more experienced recurrence, during the at least 36-month follow-up period. Patients with higher ^18^F-FDG SUVs (> 5.5) had worse OS (*p* = 0.007) and tended toward worse PFS (*p* = 0.11), while patients with lower 18F-FES SUVs (≤ 1.5) had worse PFS (*p* = 0.03) and tended toward worse OS (*p* = 0.19). Patients with ^18^F-FDG/^18^F-FES ratios > 2.6 had worse PFS (*p* = 0.009) and OS (*p* = 0.005). The 5-year PFS and OS rates were 75% and 88% for patients with lower ratios, but were only 10% and 20% for those with higher ratios. These results suggest that pretreatment tumor ^18^F-FDG/^18^F-FES ratio is useful for predicting the prognosis of uterine sarcoma patients.

## INTRODUCTION

Uterine sarcoma, which includes carcinosarcoma (CS), leiomyosarcoma (LMS), low- and high-grade endometrial stromal sarcoma (L-, H-ESS), and adenosarcoma, is a rare type of tumor that accounts for approximately 1% of female genital tract malignancies and 3%–7% of malignant uterine tumors [1, 2]. Although it might be more accurate to classify uterine CS as a metaplastic uterine carcinoma, CS is more aggressive than endometrial carcinoma and is still classified as a uterine sarcoma in most relevant retrospective studies, as well as according to the 2003 World Health Organization (WHO) guidelines [3]. Additionally, uterine sarcoma is characterized by heterogenous tumors with vastly different clinical presentations, responses to therapy, and outcomes. The prognosis of patients with uterine sarcoma is poor, with an overall 5-year survival rate of 8%–12% reported for advanced stages [3–5]. The paucity of large, randomized, controlled studies due to the rarity of this disease, as well as the heterogeneous nature of the tumors, likely contribute to the lack of effective treatments for uterine sarcoma patients.

Recent pathological analysis has revealed that a significant percentage of ESS, LMS, and CS tumors express estrogen receptors (ER) [6]. Although the precise role of hormone receptors in the disease biology of uterine sarcomas remains unclear, ER expression seems to be correlated with prognosis and response to therapy in uterine sarcomas, and hormone administration has been investigated as a treatment for uterine sarcoma [7, 8].

Unlike analysis of ER expression in tissue biopsies, ER imaging can assess heterogeneity in ER expression. Regional ER expression can be examined non-invasively using Positron Emission Tomography (PET) with 16α-^18^F-fluoro-17β-estradiol (^18^F-FES) [9]. We previously demonstrated that standard uptake values (SUV) for ^18^F-FES and ^8^F-fluoro-deoxyglucose (^18^F-FDG), as well as ^18^F-FDG/^18^F-FES SUV ratios, obtained from functional PET images reflect the expression of sex hormone receptors and tumor activity in mesenchymal uterine tumors [10–12]. However, to our knowledge, no quantitative imaging biomarkers have been established for predicting uterine sarcoma patient outcomes. Here, we investigated whether quantitative PET parameters were useful indicators of prognosis in uterine sarcoma patients with CS, LMS, and ESS histological subtypes.

## RESULTS

### Clinical and PET findings

**Figure 1:**
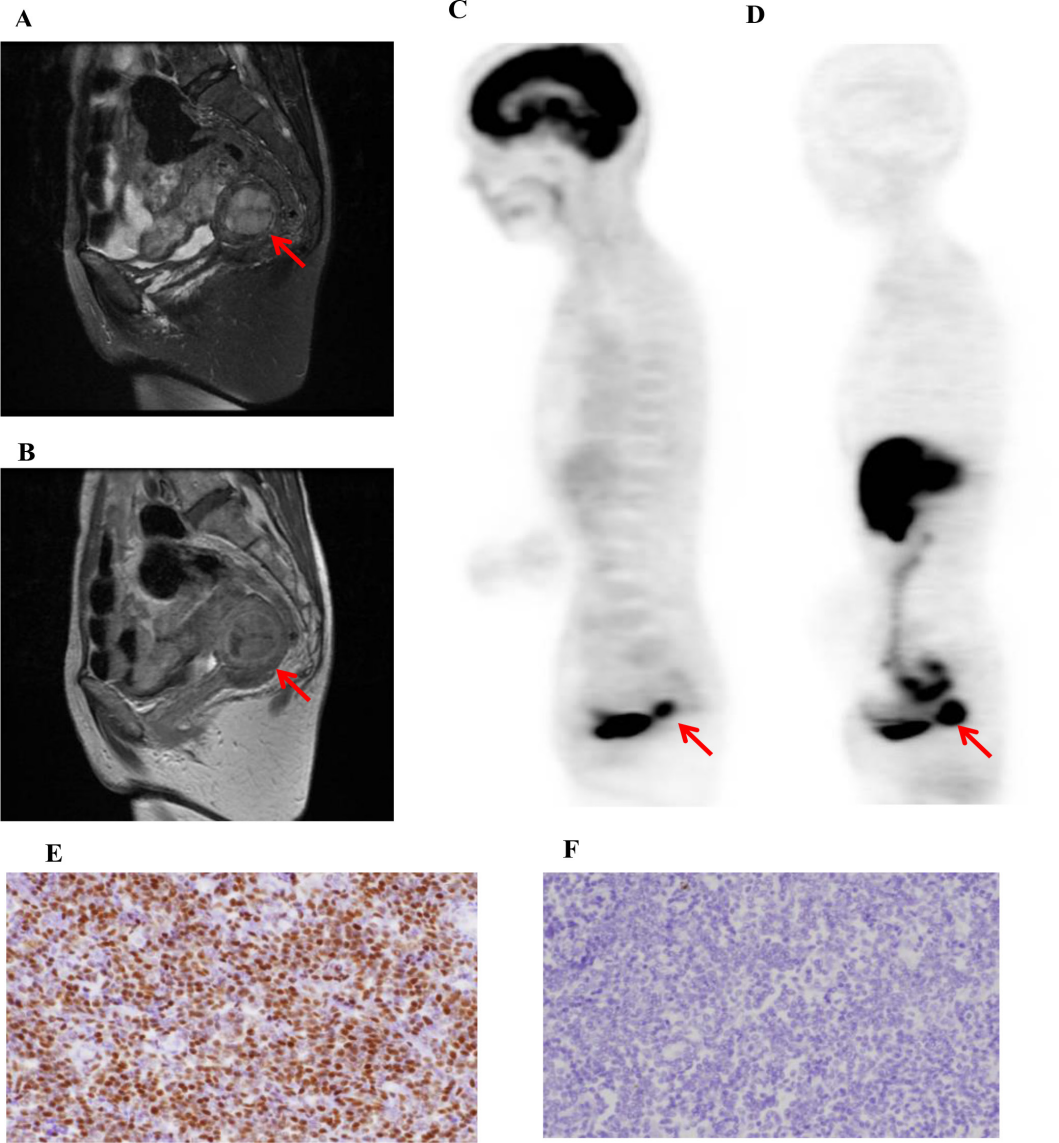
A representative case of low-grade uterine endometrial stromal sarcoma (L-ESS, 58-y-old patient, FIGO stage I) T2-weighted (**A**) and contrast-enhanced T1-weighted (**B**) MR images and ^18^F-FDG (**C**) and ^18^F-FES (**D**) PET images are shown. The ^18^F-FDG SUV, ^18^F-FES SUV, and ^18^F-FDG/^18^F-FES SUV ratio were 5.4, 12.6, and 0.4, respectively. Immunohistochemistry revealed that ER expression (**E**) was high and MIB-1 expression (**F**) was low in this case. The patient underwent an abdominal total hysterectomy with bilateral salpingo-oophorectomy (TAH + BSO) and is still alive without any recurrence or metastasis.

**Figure 2:**
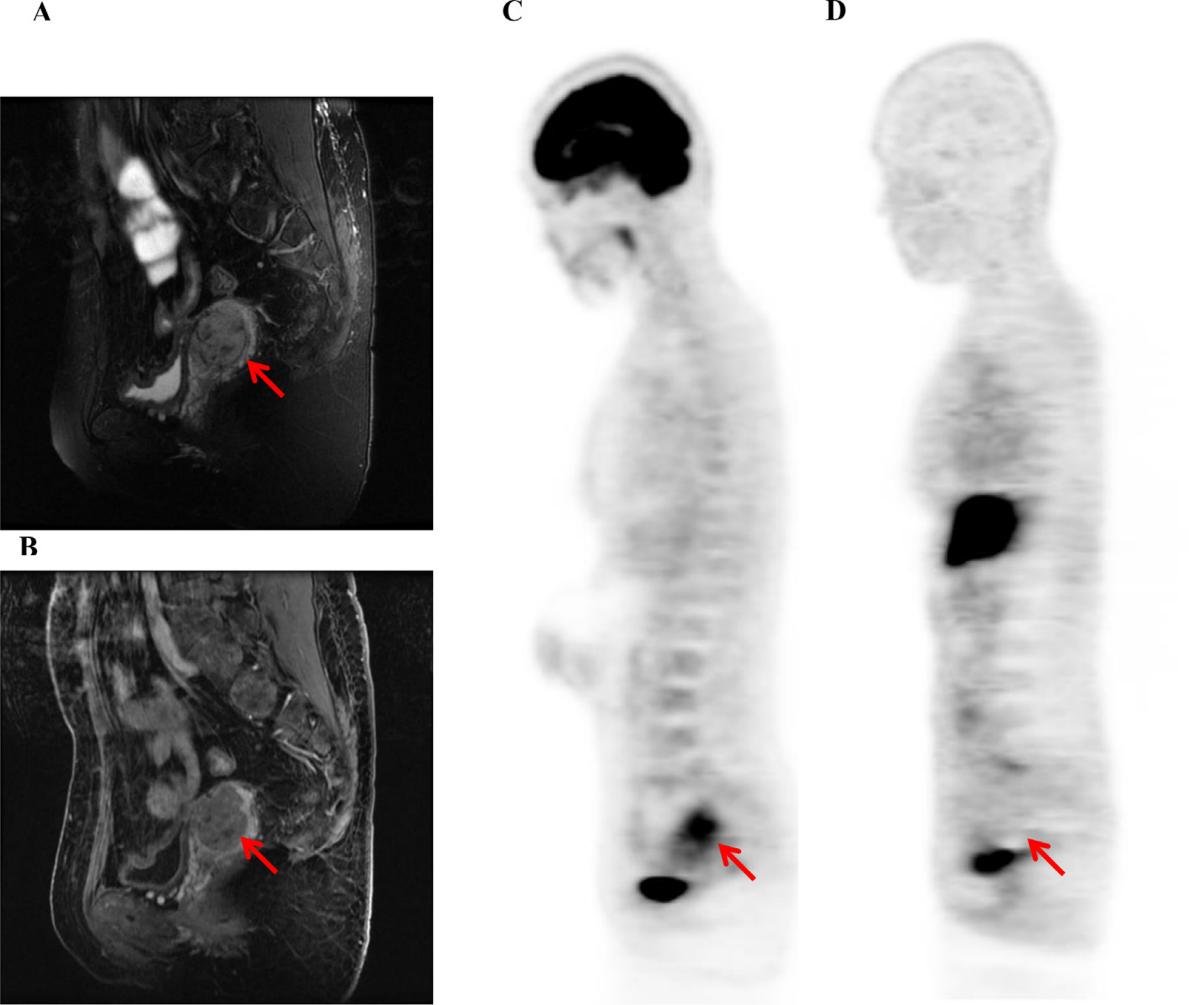
A representative case of uterine leiomyosarcoma (LMS, 41-y-old patient, FIGO stage I) T2-weighted (**A**) and contrast-enhanced T1-weighted (**B**) MR images and ^18^F-FDG (**C**) and ^18^F-FES (**D**) PET images are shown. The ^18^F-FDG SUV, ^18^F-FES SUV, and ^18^F-FDG/^18^F-FES SUV ratio were 7.0, 1.1, and 6.4, respectively. This patient also underwent TAH + BSO; however, she had local recurrence and lung metastases 2 months later and died 8 months later.

**Figure 3:**
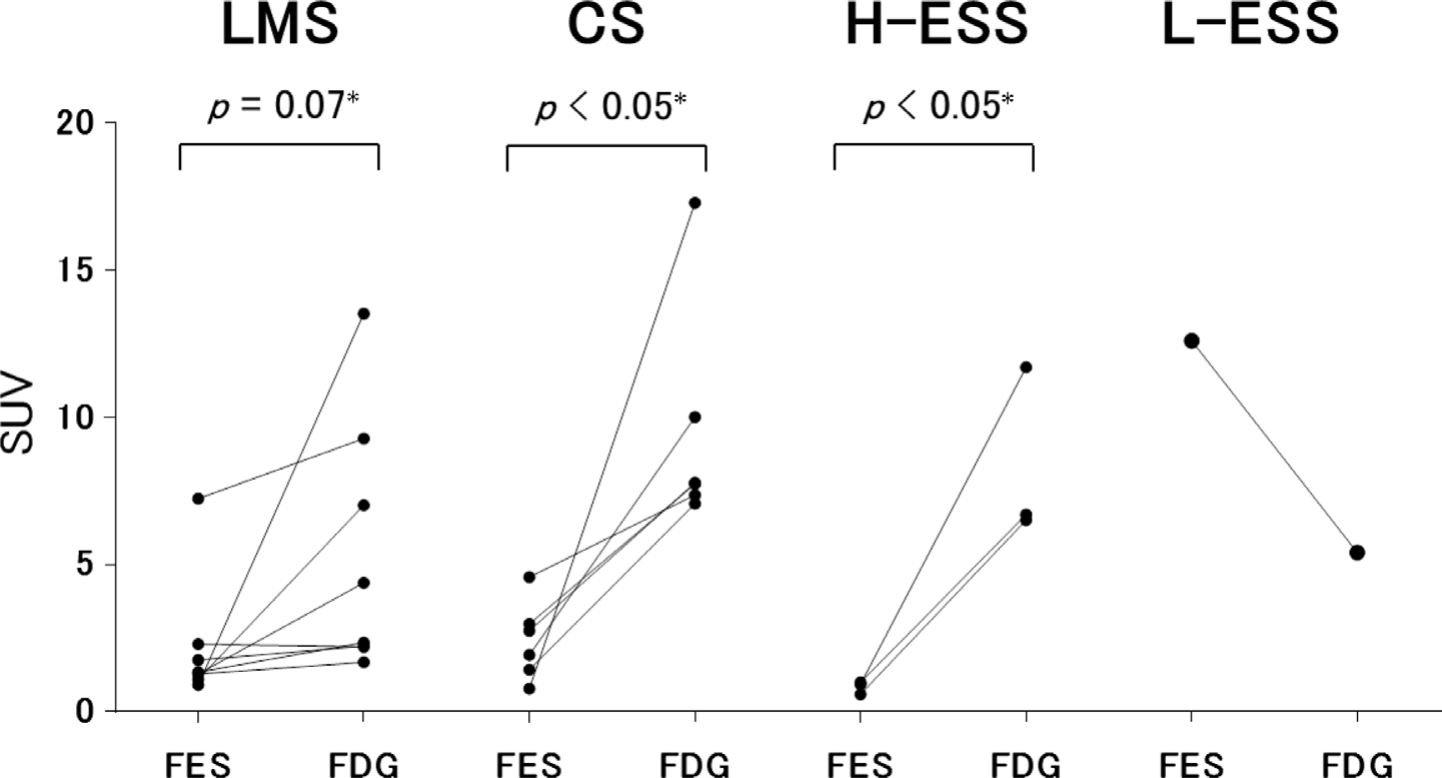
Tracer accumulation in the four histological types of uterine sarcoma Standardized uptake values (SUV) for ^18^F-FES and ^18^F-FDG in uterine sarcoma patients with leiomyosarcoma (LMS), carcinosarcoma (CS), or high- or low- grade endometrial stromal sarcoma (H-, L-ESS) are shown. Accumulation of ^18^F-FES and ^18^F-FDG were compared in each patient group. Accumulation of ^18^F-FDG was higher than that of ^18^F-FES in patients with CS or H-ESS (*p* < 0.05). There was a trend toward the same effect in patients with LMS (*p* = 0.07). In contrast, accumulation of ^18^F-FES was higher than that of ^18^F-FDG in the patient with L-ESS. Group analysis did not reveal significant differences among the four groups.

Detailed patient clinical information, such as histology type, ERα immunohistochemistry score, pathological findings, ^18^F-FES and ^18^F-FDG PET analysis, treatments, recurrence patterns, and responses to hormone therapies are summarized in Table 1. Two specimens were excluded from the analysis of ER status due to inappropriate disease state; specimens from 16 patients were evaluated. As in our previous reports, ER immunoreactive scores tended to correlate highly with ^18^F-FES uptake [10–12]. Representative cases are shown in Figures 1 and 2, and differences in tracer accumulation among the four histopathological types are shown in Figure 3. Accumulation of ^18^F-FDG was higher than that of ^18^F-FES in patients with CS and H-ESS (*p* < 0.05); there was a trend towards the same result in patients with LMS (*p* = 0.07). In contrast, accumulation of ^18^F-FES was higher than that of ^18^F-FDG in the patient with L-ESS. Group analysis did not reveal significant differences in tracer accumulation among the four groups. Patient follow-up periods ranged from 0.5 to 60 months. Ten patients died from local recurrence or metastasis, and one of the 8 remaining patients also experienced recurrence. The mean PFS and OS durations for all patients were 25.7 ± 21.3 and 33.9 ± 20.5 months, respectively.

**Table 1:** Patient characteristics according to uterine sarcoma histology

Patient	Histology	FDG SUV	FES SUV	FDG/FES ratio	ER IRS	Treatment	Recurrence pattern	Hormone therapy	PFS (m)	OS (m)
1	LMS	13.52	0.91	14.89	0	operation	-	-	60	60
2	LMS	9.28	7.24	1.28	9	operation	stump	-	16	20
3	LMS	2.35	1.34	1.75	4	operation	lung, peritoneal	-	2	60
4	LMS	4.37	1.27	3.44	2	operation	peritoneal	-	20	60
5	LMS	2.20	2.29	0.96	2	operation	-	-	60	60
6	LMS	2.22	1.75	1.27	4	operation	-	-	60	60
7	LMS	1.68	1.28	1.32	3	operation	-	-	60	60
8	LMS	7.01	1.09	6.42	4	operation	stump	-	2	8
9	CS	7.07	1.42	4.97	2	operation	lung, liver	-	1	18
10	CS	10.01	1.92	5.20	1	operation	NA	-	30	30
11	CS	7.78	2.75	2.83	0	radiation	NA	-	16	16
12	CS	7.36	4.57	1.61	0	operation	liver	-	16	37
13	CS	7.74	2.98	2.59	4	operation	-	-	39	39
14	CS	17.29	0.78	22.17	NA	chemotherapy	NA	-	0.5	0.5
15	H-ESS	6.70	0.99	6.78	1	operation	lymph node, lung, peritoneal	-	10	18
16	H-ESS	6.51	0.59	10.98	0	operation	NA	-	10	12
17	H-ESS	11.70	0.93	12.62	NA	operation	stump	-	18	30
18	L-ESS	5.41	12.6	0.43	6	operation	-	-	60	60

Abbreviations: ER IRS = estrogen receptor immunoreactive score, FES = 16α-[^18^F]-fluoro-17β-estradiol, SUV = standardized uptake value, PFS = progression-free survival, OS = overall survival, LMS = leiomyosarcoma, CS = carcinosarcoma, H-ESS = high- grade endometrial stromal sarcoma, L-ESS = low- grade endometrial stromal sarcoma, NA = not assessed, m = months.

### Survival prediction

**Figure 4:**
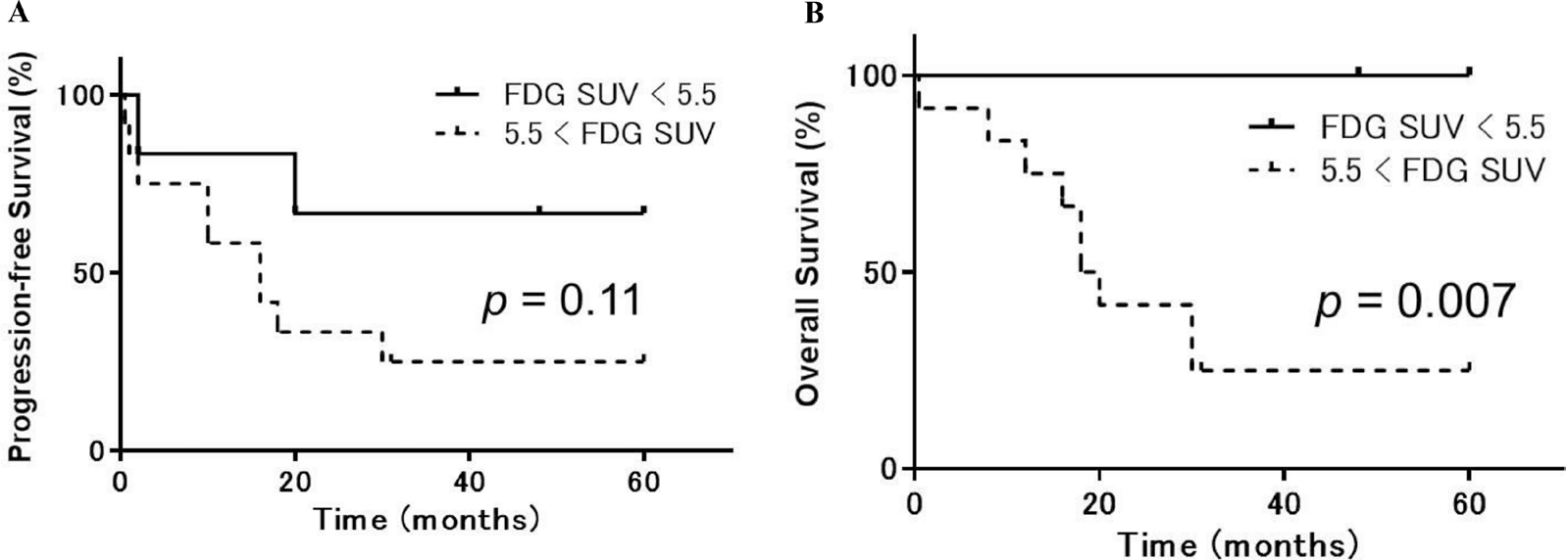
Progression-free and overall survival in uterine sarcoma patients stratified according to ^18^F-FDG accumulation Kaplan-Meier curves of progression-free survival (PFS) (**A**) and overall survival (OS) (**B**) in uterine sarcoma patients (*n* = 18). Patients were stratified based on ^18^F-FDG PET into high (> 5.5, dotted lines) or low (≤ 5.5, solid lines) ^18^F-FDG accumulation groups. Patients with higher ^18^F-FDG SUVs had worse OS (*p* = 0.007) and a trend toward worse PFS (*p* = 0.11) than those with lower ^18^F-FDG uptake.

**Figure 5:**
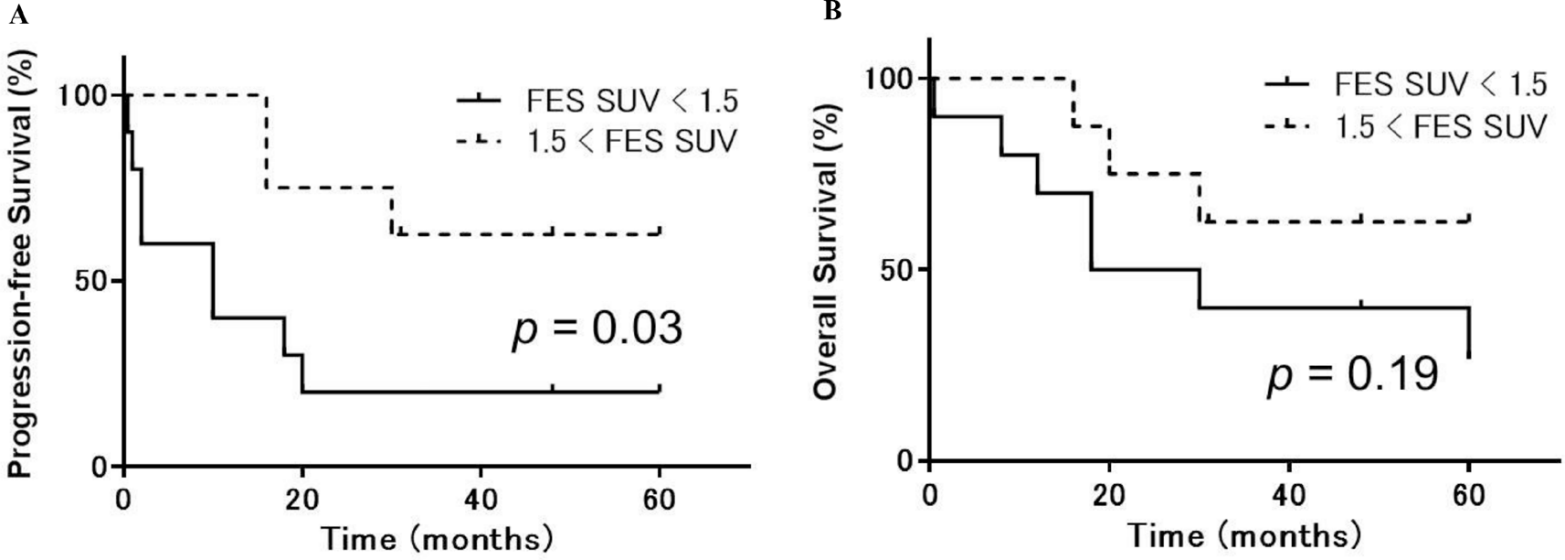
Progression-free and overall survival in uterine sarcoma patients stratified according to ^18^F-FES accumulation Kaplan-Meier curves of progression-free survival (PFS) (**A**) and overall survival (OS) (**B**) in uterine sarcoma patients (*n* = 18). Patients were stratified by ^18^F-FES PET into high (> 1.5, dotted lines) or low (≤ 1.5, solid lines) ^18^F-FES accumulation groups. Patients with lower ^18^F-FES SUVs had worse PFS (*p* = 0.03) and a trend toward worse OS (*p* = 0.19) than those with higher ^18^F-FES uptake.

**Figure 6:**
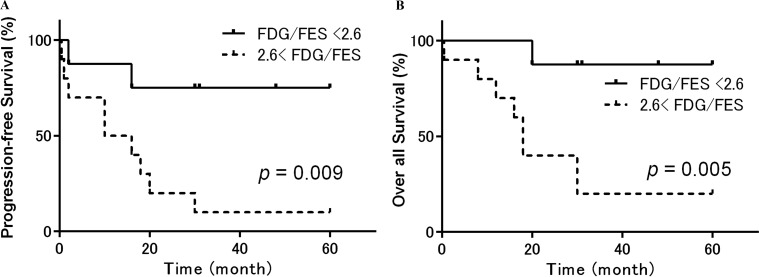
Progression-free and overall survival in patients with uterine sarcoma stratified according to the ^18^F-FDG/^18^F-FES SUV ratio Kaplan-Meier curves of progression-free survival (PFS) (**A**) and overall survival (OS) (**B**) in patients with uterine sarcoma (*n* = 18). Patients were stratified by ^18^F-FDG and ^18^F-FES PET into groups with a high (> 2.6, dotted lines) or low (≤ 2.6, solid lines) ^18^F-FDG/^18^F-FES SUV ratio. Patients with a higher SUV ratio had significantly worse PFS (*p* = 0.009) and OS (*p* = 0.005) than those with a lower SUV ratio. Five-year PFS and OS rates were 75% and 88% for patients with a lower SUV ratio (≤ 2.6); however, these rates were 10% and 20% for those with a higher SUV ratio (> 2.6).

Receiver operating characteristic analysis indicated the following optimal cutoff values for each PET index: ^18^F-FDG SUV = 5.5, ^18^F-FES SUV = 1.5, and ^18^F-FDG/^18^F-FES SUV ratio = 2.6. Using these cutoffs, patients with higher ^18^F-FDG SUVs had worse OS (*p* = 0.007) and a trend toward worse PFS (*p* = 0.11) than patients with a lower ^18^F-FDG SUVs (Figure 4). In addition, patients with lower ^18^F-FES SUVs had worse PFS (*p* = 0.03) and a trend toward worse OS (*p* = 0.19) than patients with higher ^18^F-FES SUVs (Figure 5). Using the specified cutoff value for the ^18^F-FDG/^18^F-FES SUV ratio, patients with higher SUV ratios had worse PFS (*p* = 0.009) and OS (*p* = 0.005) than those with lower SUV ratios (Figure 6). Five-year PFS and OS rates were 75% and 88% for patients with lower SUV ratios (≤ 2.6); in contrast, these rates were 10% and 20% for those with higher SUV ratios (> 2.6). Univariate analyses using a Cox proportional hazard regression model revealed that ^18^F-FDG/^18^F-FES SUV ratio and OS were correlated (*p* = 0.03), and ^18^F-FDG/^18^F-FES SUV ratio and PSF were strongly correlated (*p* = 0.051).

## DISCUSSION

We found that uptake ratio of the non-invasive molecular image biomarkers ^18^F-FDG and ^18^F-FES was useful for predicting uterine sarcoma patient outcomes. Despite heterogeneity in treatment regimens, disease stages, and histological types in the patients examined in this retrospective study, ^18^F-FDG/^18^F-FES SUV ratio was correlated with both PFS (*p* = 0.007) and OS (*p* = 0.005). With a few exceptions, PET revealed that the accumulation of ^18^F-FES in disease sites of patients with high ^18^F-FDG accumulation was low. These results are similar to those of our previous ^18^F-FES PET uterine sarcoma studies [14–16]. Furthermore, qualitative PET findings revealed that a large fraction of patients showed low ^18^F-FES uptake and, importantly, that low ^8^F-FES uptake tended to predict poor prognosis in these patients; however, this effect was not statistically significant, likely due to the small number of samples included in the study. In contrast, high ^18^F-FES uptake was observed in the one L-ESS patient, who also had a good prognosis. Likewise, LMS patients also had relatively high ^18^F-FES uptake and good prognoses.

Our previous report using immunohistochemical analysis indicated that ^18^F-FES uptake was associated with ERα immunohistochemistry score [12]. Although the functional significance of ER expression in uterine sarcoma is unclear, previous immunohistochemical analyses indicate that it is expressed in 70–75% of L- and H-ESS, 50–60% of LMS, and 30–35% of CS specimens [6, 7]. In addition, Ioffe *et al*. reported that patients with ER-positive uterine sarcomas had longer overall survival compared to patients with ER negative sarcomas [7].

Increasing evidence has indicated that, in addition to ERα, the expression of PR and PR-B may also be associated with prognosis in uterine sarcoma. A previous study using immunohistochemistry indicated that the expression of ERα and PR and of ERα and PR-B were correlated in mesenchymal uterine tumors [12]. Furthermore, ERα activation induces PR expression [13]. Additionally, PR expression seems to be indicative of cases in which cancer is confined to the uterine body, which have better outcomes. Leitao *et al*. reported that ER/PR expression is associated with survival outcomes in patients with high-grade uterine LMS confined to the uterine body [6]. In a phase 2 trial of aromatase inhibition in uterine LMS patients, the disease control period of more than 24 weeks for tumors in which > 90% of cells expressed both ER and PR was longer than that observed in patients with lower ER and PR expression [8]. Koivistro-Korander *et al*. also demonstrated that expression of these hormone receptors was associated with therapeutic outcomes; ER/PR-positive ESS, LMS, and CS patients tended to have longer PFS and OS [14]. Recently, Yoon *et al*. assessed prognostic factors associated with disease-related survival using the 2009 FIGO staging system and found that stage, ER/PR expression, and nodal metastasis are associated with OS in ESS patients [15].

Although immunohistochemistry assays are less expensive than performing two PET scans, the pathological assays used for ERα have some limitations because the receptor antigen may be present even if the ERα is nonfunctioning. Furthermore, it is not possible to evaluate the status of the entire tumor using immunohistochemistry for ERα, especially considering tumor heterogeneity. In addition, immunohistochemistry assays cannot provide information about regions for which surgical biopsies are not possible [16–18]. Because none of the patients examined here were treated using hormone therapy, we could not evaluate correlations between tumor ^18^F-FES uptake and responses to hormone therapy. However, ^18^F-FES uptake measurements might assist in predicting response to hormone therapy in uterine sarcoma patients, while immunohistochemistry assays might not.

Currently, ^18^F-FDG is widely used to evaluate regional glucose metabolism in a variety of tumors, including uterine sarcomas [19, 20], and high correlations have been found between ^18^F-FDG uptake and GLUT-1 expression in most malignant tumors [21–23]. We previously reported that ^18^F-FDG SUV was correlated with GLUT-1 expression in ovarian and mesenchymal uterine tumors [12, 21]. Although GLUT-1 expression may be a major cause of high ^18^F-FDG uptake in uterine tumors, other factors, such as expression of hexokinase II, tumor cell proliferation, hormonal dependency, microvessel density, and the presence of inflammatory cells, might also contribute to high uptake [23]. Because it is influenced by many factors, it would likely be difficult to use ^18^F-FDG SUV alone as a prognostic tool for patients with mesenchymal uterine tumors.

In our previous study, the ^18^F-FDG/^18^F-FES SUV ratio was negatively correlated with ERα, PR, and PR-B expression and positively correlated with Ki-67 LI in total mesenchymal uterine tumors. ^18^F-FDG/^18^F-FES ratio was also positively correlated with ERβ and GLUT-1 expression in sarcoma patients. The ^18^F-FDG/^18^F-FES ratio, which reflects glucose metabolism relative to ERα density, correlated better with the Ki-67 index in uterine sarcoma than did either ^18^F-FDG or ^18^F-FES SUV alone [12]. We therefore suggest that, as an index coupling ER expression and glucose metabolism, the ^18^F-FDG/^18^F-FES SUV ratio might be a useful indicator of the relationship between sex hormone receptor status and cell proliferation in uterine tumors, especially in uterine sarcoma. Although we did not directly assay mitotic index in the present study, several prior studies indicated that proliferation biomarkers, such as Ki-67 and MIB-1, predicted recurrence [24, 25].

Our results indicate that SUVs and the ^18^F-FDG/^18^F-FES SUV ratio determined from PET images are a potential prognostic marker in patients with various histological subtypes of uterine sarcoma, including CS, LMS, and ESS. However, the number of patients included in this study is small, and they differed substantially in histopathologic type. Although these aspects may represent limitations of the current study, PET imaging helped to reduce overlap in prognosis among the stages of the 2009 FIGO staging system, and most of the cases examined here were classified as stage I disease, with very few stage II or III cases. PET imaging may therefore aid in predicting differences in prognosis among patients with stage I sarcoma in general, regardless of the histological type. Multicenter collaborative investigations are needed to confirm the utility of PET images for guiding treatment decisions for uterine sarcoma patients.

In summary, PET molecular imaging using ^18^F-FES, which assesses ERα expression, and quantitative measurement of the ^18^F-FDG/^18^F-FES SUV ratio may be useful for predicting outcomes in uterine sarcoma patients. These measurements might therefore assist in the personalized management of these patients and could improve their prognosis.

## MATERIALS AND METHODS

### Patients

In this retrospective analysis, we evaluated eighteen patients with a histological diagnosis of uterine sarcoma (mean age = 58.9 years) who had undergone preoperative ^18^F-FES and ^18^F-FDG PET scans at the University of Fukui Hospital between August 2005 and August 2013. Histopathological examinations were performed on surgical specimens for 17 of the patients and on a biopsy specimen for one patient. Eight patients were diagnosed with LMS, 6 with CS, 3 with H-ESS, and one with L-ESS. Patient follow-ups were conducted for at least 36 months or until death. The study protocol was approved by the ethics committee of the Faculty of Medical Sciences, University of Fukui, and informed consent was obtained from all patients before PET scanning.

### PET procedure

^18^F-FES was synthesized as previously described [9]. In brief, 3-*O*-methoxymethyl-16β,17β-*O*-epiestriol cyclic sulfone was fluorinated, and 2-step hydrolysis and neutralization were then performed using a TRACERlab MX_FDG_ (GE Healthcare). After the final purification, the specific activity was 100–200 GBq/μmol, and radiochemical purity was greater than 99%. The radiochemical yield was 16.6% ± 3.0% at the end of synthesis.

^18^F-FES PET scans were obtained using a whole-body PET scanner (Advance; GE Medical Systems, Milwaukee, WI) that permits simultaneous acquisition of 35 image slices in a 2-dimensional acquisition mode with an interslice spacing of 4.25 mm. Performance tests showed that the intrinsic resolution of the scanner was 4.0–5.3 mm in the axial direction and 4.6–5.7 mm in the transaxial direction. ^18^F-FDG PET scans were obtained using either the Advance scanner or a combined PET/CT scanner (Discovery LS; GE Medical Systems, Milwaukee, WI) with the same acquisition capabilities. The PET/CT scanner incorporated an integrated four-slice multidetector CT scanner that was used for attenuation correction.

Two PET scans were performed on two separate days within one week in a random sequence. For each ^18^F-FES and ^18^F-FDG PET scan, approximately 185 MBq of the tracer were administered via the antecubital vein. Patients fasted for at least 4 h before each study. Fifty minutes after tracer injection, the patient was scanned in the PET or PET/CT scanner in a supine position. For PET, a 16-min emission scan was obtained, with 3-min scans in the pelvic region (2 bed positions) and 2-min scans in each remaining region (5 bed positions) for complete coverage from the head to the inguinal area. Post-injection transmission scans with 2 min durations at the pelvis and 1 min in other areas were acquired after the emission scans using a ^68^Ge/^68^Ga rod source for attenuation correction. CT scanning parameters for attenuation correction of PET/CT were as follows: auto mA (upper limit, 40 mA; noise index, 20), 140 kV, 5-mm section thickness, 15-mm table feed, and pitch of 4. After the CT transmission scan, a whole-body emission scan was performed from the head to the inguinal region at 2 min per bed position (seven to eight bed positions). PET data were reconstructed using the iterative reconstruction method with 14 subsets and 2 iterations. The reconstructed images were then converted into a SUV.

### Image analysis

All patients underwent MR imaging before the 2 PET examinations for diagnosis and to obtain anatomic information about the pelvic organs. T1- and T2-weighted images in the axial, sagittal, and coronal planes were acquired using 1.5-T or 3.0-T superconducting MR imaging systems (Signa; GE Medical Systems, Milwaukee, WI). Contrast-enhanced MRIs were obtained with or without fat saturation for the axial and sagittal planes after the injection of gadolinium diethylenetriamine pentaacetic acid (0.1 mmol/kg).

Images were analyzed as previously reported [10–12]. Multiple circular regions of interest (ROIs) with fixed diameters of 8 mm were drawn on the lesions to obtain regional mean SUVs in the ROIs. Individual MR images were referenced to ensure that ROIs were placed in the appropriate regions after PET and MR images were coregistered using software (Body Guide, Advance Biologic Co., Toronto, Canada). ROIs were applied to the resliced ^18^F-FES and ^18^F-FDG PET images in the same location because the 3 images had the same space coordinates. Two or 3 sagittal or coronal planes of 6-mm thickness were used to obtain SUVs at the center of the lesion. If the center of the lesion was necrotic, ROIs were placed so as to exclude the necrosis on the MR image. SUVs for each patient were averaged for all ROIs to obtain mean ^18^F-FES and ^18^F-FDG SUVs for the tumor. The ^18^F-FDG/^18^F-FES SUV ratio for each lesion was also calculated.

### Immunohistochemistry

Immunohistochemistry was performed as previously described [12] using paraffin sections (2.5 mm thick) and a standard immunohistochemistry technique (avidin–biotin–peroxidase). A primary antibody against ERα (monoclonal mouse, 1D5, Abcam, 1:150) was used. Sections of human colon and breast cancer were used as positive controls, and negative controls were obtained by omitting the primary antibody. The intensity and distribution of nuclear ERα receptor staining were semi-quantitatively assessed using an immunoreactive score (IRS) as previously described [12]. The immunoreactive score (range, 0–12) was calculated according to the following equation: IRS = ΣSI x PP, where SI is the optical staining intensity, graded as 0 = none, 1 = weak, 2 = moderate, or 3 = strong, and PP is the percentage of positively-stained cells, defined as 0 = no staining, 1 ≤ 10%, 2 = 11%–50%, 3 = 51%–80%, and 4 ≥ 81%.

### Statistical analysis

The mean SUVs of the lesions and their ratios were compared among the four groups (LMS, CS, H-ESS, and L-ESS) using analysis of variance. Differences in accumulation of the two tracers were also compared using paired *t*-tests.

Progression-free survival (PFS) and overall survival (OS) served as the outcome measures. PFS was defied as the time from initiation of treatment to locoregional or systemic recurrence, or to cancer death. Study participants were staged according to the 2009 International Federation of Gynecology and Obstetrics (FIGO) classifications. PET results were correlated with clinical follow-up data, and Receiving Operating Characteristics (ROC) analyses were performed to determine optimal cut-off values for dividing patients with and without events (disease progression or death) at the time of the last post-treatment follow-up. PFS and OS rates were estimated using the Kaplan-Meier method and were compared using the log-rank test and univariate Cox regression.

All statistical analyses were performed using SPSS Statistics Version 22 (IBM, Armonk, NY, USA). A probability of less than 0.05 was considered statistically significant.
